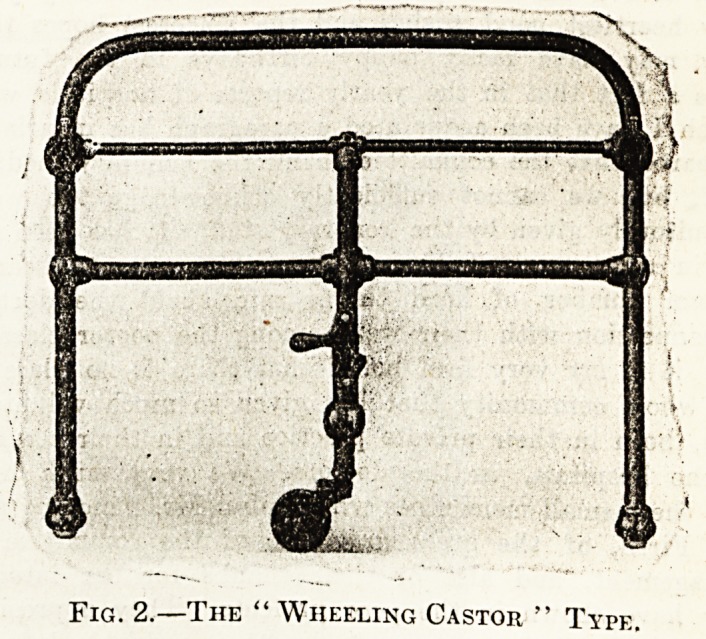# Two Bed-Lifters Described

**Published:** 1914-05-23

**Authors:** 


### TWO BED-LIFTERS DESCRIBED.

The bed-lifter shown in Fig. 1, which Messrs. Whitfields Bedsteads Ltd., of Bordesley, Birmingham, have been making for many years, is used for lifting the foot-end of a bedstead for the purpose of moving the bedstead from place to place; the makers describe it as very easily detachable and very easily applied. At the top of the lifter there are two hooks (one at each side) which are passed under the foot-bow, and by lifting the top of the lifter the bottom part of it inclines right up towards the bedstead, and on the upright of the lifter at each side there are movable buttons which are then turned down over the crossbar below seat at foot-end of bedstead.

Fig. 1.—The Detachable Type.

This is used on bedsteads which are constructed with castors on the head-legs, and with wood feet on the foot-legs, bedsteads being so constructed that when they are in occupation they will not move while the patient is being attended to or if anybody knocks against the bedstead in passing.

Until this bed-lifter was produced it was necessary for the attendant to lift the whole foot-end of the bedstead with the patient in it in order to wheel the bedstead from place to place, but the introduction of this bed-lifter for use with the "Lawson Tait" bedsteads, makes it quite a simple matter for the attendant to move the bedstead easily from one place to another, and when removed the bed-lifter is detached from the foot-end and can be used for another bedstead.

Fig. 2.—The "Wheeling Castor" Type.

Messrs. Whitfields also make a "wheeling castor," illustrated in Fig. 2. This shows a castor mounted on a column in the centre of the foot-end, and made so that it can be automatically brought into use by just raising the foot-end of the bed about two inches, and then the bedstead can be easily wheeled from place to place. The castor is easily put out of use by slightly raising the handle; this action lets the foot-end down on to the wooden feet on each leg.

## Figures and Tables

**Fig. 1. f1:**
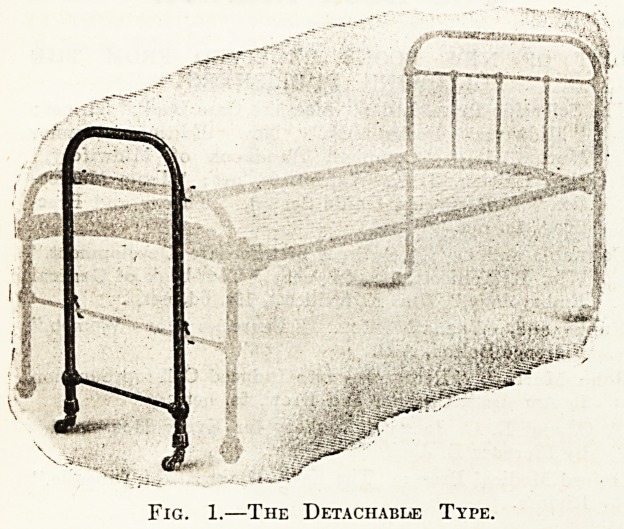


**Fig. 2. f2:**